# Level and intensity of objectively assessed physical activity among pregnant women from urban Ethiopia

**DOI:** 10.1186/1471-2393-12-154

**Published:** 2012-12-17

**Authors:** Mads F Hjorth, Stine Kloster, Tsinuel Girma, Daniel Faurholt-Jepsen, Gregers Andersen, Pernille Kæstel, Søren Brage, Henrik Friis

**Affiliations:** 1 Department of Nutrition, Exercise and Sports, Faculty of Science, University of Copenhagen, Rolighedsvej 30, DK-1958, Frederiksberg, Denmark; 2 Department of Paediatrics and Child Health, Jimma University Specialized Hospital, Jimma, Ethiopia; 3 Steno Diabetes Center, Gentofte, Denmark; 4 Medical Research Council Epidemiology Unit, Institute of Metabolic Science, Cambridge, U.K

**Keywords:** Accelerometry, Heart rate, Low-income countries, Physical activity, Pregnancy

## Abstract

**Background:**

Women in low-income countries are generally considered to have a high physical workload which is sustained during pregnancy. Although most previous studies have been based on questionnaires a recent meta-analysis of doubly labeled water data has raised questions about the actual amount of physical activity performed. In this study we report objectively assessed levels of physical activity, cardiorespiratory fitness and muscular fitness among pregnant urban Ethiopian women, and their association with demographic characteristics and anthropometric measures.

**Methods:**

Physical activity was measured for seven consecutive days in 304 women using a combined uniaxial accelerometer and heart rate sensor. Activity energy expenditure was determined using a group calibration in a branched equation model framework. Type and duration of activities were reported using a 24-hour physical activity recall and grip strength was assessed using a dynamometer.

**Results:**

Median (interquartile-range, IQR) activity energy expenditure was 31.1 (23.7-42.0) kJ/kg/day corresponding to a median (IQR) physical activity level of 1.46 (1.39-1.58). Median (IQR) time in sedentary, light, and moderate-to-vigorous intensity was 1100 (999–1175), 303 (223–374) and 40 (22–69) min/day, respectively. Mean (standard deviation) sleeping heart rate was 73.6 (8.0) beats/min and grip strength was 21.6 (4.5) kg. Activity energy expenditure was 14% higher for every 10 cm^2^ difference in arm muscle area and 10% lower for every 10 cm^2^ difference in arm fat area and 10-week difference in gestational age.

**Conclusion:**

The level and intensity of physical activity among pregnant women from urban Ethiopia is low compared to non-pregnant women from other low income countries as well as pregnant European women from high-income countries.

## Background

Women in low-income countries are generally considered to have a high physical workload [1-4], which is sustained during pregnancy [2,5], and may contribute to the high incidence of low birth weight [5-7]. However, there are only few published studies on physical activity among pregnant women in low-income countries, and most have been based on questionnaires. In a recent meta-analysis it was suggested that women from developing countries perform similar amounts of physical activity as women from developed countries when assessed by doubled labeled water [8] raising questions about the actual physical workload. However, these data provide no insight into the patterns of physical activity. Thus, there is a need for more studies with objective methods for assessing physical activity among pregnant women in low-income countries.

Combined heart rate (HR) and movement sensing has recently been shown to provide valid estimates of free-living physical activity [9]. This objective method has also been applied to pregnant European populations [10,11] but to our knowledge, never to a pregnant population in a low-income setting. Using objective methods during pregnancy is important to improve the understanding of the level and predictors of physical activity, and may provide information to improve future maternal and child health in low-income countries.

In this paper, we report objectively assessed levels of physical activity, as well as cardiorespiratory- and muscular fitness among urban pregnant women in Ethiopia. Furthermore, we report associations between these measures and demographic characteristics and anthropometric measurements.

## Methods

### Study area and population

This cross-sectional study was conducted in Jimma, Ethiopia, between December 2008 and August 2009. Jimma is situated in the Oromia region at an altitude of 1750 m above sea level, 350 km south west of Addis Ababa, and is the largest city in south-western Ethiopia with a population of approximately 120,600 [12]. Pregnant women living in Jimma town were invited to participate in the study during their routine visits at the antenatal care clinic, Jimma University Specialized Hospital. If they agreed to participate, a trained nurse administered a background questionnaire and undertook anthropometric measurements, followed by a self-paced walk test and habitual physical activity assessment using a combined heart-rate and activity monitor. After 7 days, the women returned to the ANC, where the free-living assessment was terminated. The four nurses engaged in the present study were experienced Ethiopian nurses (≥5 years) from the local community. All spoke good Amheric, Afar Oromo and English, and were extensively trained for several weeks prior to the study in all relevant procedures. Throughout the study, daily communication between the nurses and the study coordination team was set up to assist and ensure high quality of data. Participating women received a reimbursement of 5 birr (≈0.50 USD) to cover transportation expenses. Women who were seriously ill or unable to walk unassisted were excluded from the study. All women received oral and written information about the study in their local language before giving written consent. Permission to conduct the study was given by the Ethical Review Board at Jimma University (RPO/86/2001) and approved by the Danish National Committee on Biomedical Research Ethics (J.nr.:2008-7041-126).

### Assessment of habitual physical activity and cardiorespiratory fitness

Physical activity was assessed using a combined uniaxial accelerometer and HR sensor (Actiheart 4, CamNtech Ltd, Papworth, UK). The monitor was placed on the left side of the trunk, at the level of the third intercostal space, on two ECG electrodes (Unilect TM Diagnostic ECG Ref 0668M, Redditch, UK); one electrode on the sternum and one as lateral as possible without stretching the wire [13]. The skin was gently cleaned before attachment. For individual calibration of heart rate, a self-paced walk test was performed. Participants were instructed to walk 250 m on level ground at their normal walking pace. Walk speed (m/min), walk HR above sleep (beats/min) and recovery HR during the first 90 s after completion (beats/min) were derived as proxy measures of cardiorespiratory fitness. Walk speed (m/min) and walk HR above sleep (beats/min) were combined into speed over ratio of HR (m/beat).

After the walk test, the monitor was initialized to collect HR and acceleration at a time resolution (epoch) of 15 seconds and the participants were asked to wear it for seven consecutive days, while continuing their normal daily activities. Two sets of additional ECG electrodes were provided along with instructions on how to replace them if necessary. At the end of the observation period, participants returned the monitor to the clinic and the data were downloaded to a computer for further processing. For each individual, the free-living recording was manually trimmed to the exact time point where the monitor had been detached. Sleeping HR was determined as the average of the non-noisy daily HR minima across all measured days except the first.

HR data was pre-processed using Gaussian Process Regression for inferring the latent HR from potentially noisy HR data [14]. Prolonged periods of no movement combined with non-physiological HR data were flagged as non-wear. The estimation of activity intensity from HR was determined using a group calibration [15] derived on the basis of >1000 step tests from a population study in Kenyan adults [16].

Activity energy expenditure (AEE) was estimated using branched equation modeling [17] to combine group calibrated HR with an acceleration based estimate of intensity [15], which was then integrated with respect to time to yield a daily average AEE (kJ/kg/day). Participants having less than 24 hours of valid recording were excluded.

Resting metabolic rate (kJ/kg/day) was estimated using age, sex, weight, and height [18], to which AEE was added and this sum divided by 0.9 to derive total energy expenditure (kJ/kg/day), under the assumption that diet-induced thermogenesis amounts to 10% of total energy expenditure. Physical activity level (PAL) was calculated as the ratio of total energy expenditure and resting metabolic rate. The distribution of physical activity intensity was summarized in three different categories of metabolic equivalence of task (MET) based on the time spent (min/day) in each category; i.e. sedentary (≤1.5 MET), light (1.5-3 MET), and moderate-to-vigorous activity (>3 MET).

### Anthropometry, body composition and muscular fitness

Barefooted and wearing light clothes, the women were weighed to the nearest 0.1 kg (Tanita Model 418, Tokyo, Japan) and height measured to the nearest 0.1 cm (Seca 217, Hamburg, Germany). On the left arm, mid-upper-arm circumference (MUAC) was measured to the nearest 0.2 cm using a Small Insertion Tape (Product code A/IT, TALC, St Albans, UK) and triceps skinfold thickness (TSF) measured in duplicate to the nearest 0.2 mm using a Harpenden Skinfold Calliper (Model HSB-BI, Baty International, West Sussex, UK). From these measurements, body mass index (BMI) was computed as weight/height^2^ (kg/m^2^), and arm fat area (AFA) as (TSF [cm] x MUAC [cm]/2) – (π x TSF^2^ [cm]/4) and arm muscle area (AMA) as (MUAC [cm] – (π x TSF [cm])^2^)/4π [19]. Muscular fitness (grip strength) was measured to the nearest 0.1 kg using a grip strength dynamometer (T.K.K 5401 Grip-D, Takei Scientific Instruments CO., LTD, Niigata, Japan). Two measurements were taken from each hand, with the mean of the highest measurement from each arm displayed on the dynamometer. This procedure was repeated twice and the higher value reported.

### Questionnaire

Women were interviewed in their local language about age, education, occupation, gravidity, gestational age, and marital status. Gestational age was estimated based on first day of last menstrual period, as written in the antenatal card or reported by the woman. If this was not available, the woman was asked about the duration of her pregnancy. A 24-hour physical activity recall was conducted by a trained nurse to obtain information on the type and duration of activities performed by the women the previous day. The recall was done when the women returned the activity monitor to the clinic. The activities reported were grouped into eating, sitting, walking, sleeping / resting in bed, cooking, cleaning and outdoor work regardless of time and place of the activity. Time spent walking while carrying loads were recorded as outdoor work.

### Data analysis

Data were double-entered, compared, and corrected in EpiData Entry 3.1 (The EpiData Association, Odense, Denmark) and analyzed in STATA/IC 10.1 (Houston, US). Gravidity was categorized as primigravida, secundigravida, and multigravida. Ethnicity, religion and occupation were all grouped into three categories – the two most common and an “other” category. Education was dichotomized into having a maximum of some primary education or having finished primary education. Gestational age was analyzed both as a continuous variable and dichotomized into before and after week 28 (start of 3rd trimester).

Descriptive characteristics of the study sample are presented as means and standard deviation (SD), median and inter-quartile range (IQR) or as proportions. In association analyses, results are presented as means and 95% confidence interval (CI). AEE was positively skewed and therefore log-transformed. Simple regression analysis was used to identify potential predictors of sleeping HR, grip strength, and AEE; however AEE was adjusted for age and fitness (speed over ratio of HR in the walk test, m/beat) in all analyses. The multiple regression models for AEE, sleeping HR, and grip strength were performed using backward elimination including selected predictor variables only. Due to collinearity only age, gestational age, height, AFA and AMA were selected for backward elimination. From the multiple model, linearity between residuals and each of the dependent variables in the model, normal distribution of the residuals, as well as homogeneity of variance of the residuals were visually checked. The level of significance was considered at p<0.05.

## Results

Among 374 eligible women invited to participate in the study, 81% (n=304) had complete activity data and were included in the analyses. Reasons for not being included were refusal (n=40), incomplete physical activity data due to missing activity recordings (n=11), or observation period lasting less than 24 hours (n=19).

Background characteristics and anthropometric measurements of the participants are presented in Table 1. The women had a median (IQR) age of 23 (20–27) years. Their gestational age was between 7 and 40 weeks; with 4.7%, 58.5%, and 36.8% being in first, second and third trimester of pregnancy, respectively. Most women were either married or cohabitating (96.4%), with 52.3% being housewives, 33.9% being employed, and 13.8% being unemployed or students. Of the women participating in the first, second and third trimester of their pregnancy, 35.7, 7.4, and 4.5% had a BMI<18.5 kg·m^-2^, respectively.

**Table 1:** Background characteristics and anthropometric measurements among 304 pregnant Ethiopian women

**Variable**	**n**	**Mean (SD), median (range) or proportions (%)**	
Age (years)	304	23	(16;45)
Gravidity (number)	304	2	(1;8)
1	118	38.8%	
2	90	29.6%	
≥3	96	31.6%	
Gestational age (weeks)	299	25.0	(7.3)
Education	304		
None or some primary schooling	133	43.8%	
Completed primary school	171	56.3%	
Weight (kg)	304	55.8	(8.2)
Height (cm)	304	158.0	(5.8)
Body mass index (kg·m^-2^)	304	22.3	(2.8)
Triceps skinfold thickness (mm)	304	14.6	(5.5)
Mid upper arm circumference (cm)	304	25.1	(2.8)
Arm fat area (cm^2^)	303	17.0	(7.7)
Arm muscle area (cm^2^)	303	33.8	(5.5)

The complete time reported in the 24-hour physical activity recall is presented in groups of common activities in Table 2. Median (IQR) time spent in sedentary activities (eating, sitting and sleeping / resting in bed) and potentially strenuous work (cleaning and outdoor work) were 410 (273;538) and 30 (0;73) min/day, respectively.

**Table 2:** Type of activities reported by 288 pregnant Ethiopian women using a 24-hour physical activity recall

**Activities (min)**	**Mean (SD) or median (IQR)**	
Total daytime activities reported	659	(164)
Eating	50	(35;75)
Sitting	215	(80;375)
Sleeping / resting in bed	90	(0;180)
Walking^1^	40	(0;113)
Household activities	140	(60;248)
Cooking	90	(30;150)
Cleaning	20	(0;60)
Outdoor work^2^	0	(0;0)

^1^Walking includes transport, recreation etc. Walking carrying loads is included in outdoor work.

^2^17% of the women were engaged in outdoor work.

The activity monitor was worn for a median (IQR) duration of 6.7 (4.4-7.0) days. Physical activity and fitness measurements are presented in Table 3. Median (IQR) AEE was 31.1 (23.7-42.0) kJ/kg/day corresponding to a median (IQR) PAL of 1.46 (1.39-1.58). Women spent a median of 1100 min/day (76.4%) in sedentary activities (≤1.5 MET), 303 min/day (21.0%) in light activity intensity (1.5-3 MET), and 40 min/day (2.8%) in moderate-to-vigorous intensity (>3 MET). Mean sleeping HR was 73.6 (8.0) beats/min and grip strength was 21.6 (4.5) kg.

**Table 3:** Physical activity and fitness measures in 304 pregnant Ethiopian women

**Variable**	**n**	**Mean (SD) or median (IQR)**	
Habitual Physical Activity and Energy Expenditure			
Activity energy expenditure (kJ/kg/day)	304	31.1	(23.7;42.0)
Activity energy expenditure (kJ/day)	304	1680	(1297;2239)
Resting metabolic rate (kJ/day)^1^	304	5337	(449)
Physical activity level	304	1.46	(1.39;1.58)
Sedentary activity intensity (≤1.5 MET) (min/day)	304	1100	(999;1175)
Light activity intensity (1.5-3 MET) (min/day)	304	303	(223;374)
Moderate and vigorous intensity (>3 MET) (min/day)	304	40	(22;69)
Fitness indicators			
Sleeping heart rate (beats/min)	304	73.6	(8.0)
Walking speed (m/min)	300	66.7	(7.5)
Walking heart rate above sleep during walk (beats/min)	298	37.2	(11.0)
Walking speed / walking heart rate above sleep (m/beat)	297	1.83	(1.52;2.22)
90-sec recovery heart rate above sleep (beats/min)^2^	252	26.9	(11.0)
Grip strength (kg)	303	21.6	(4.5)

^1^Resting metabolic rate is estimated based on age, sex, height and weight [18].

^2^Heart rate signal was lost in the recovery period for n=48 women.

The predictors of AEE, sleeping HR, and grip strength are presented in Table 4 and Table 5, based on simple and multiple regression analyses, respectively.

**Table 4:** Potential predictors of activity energy expenditure (kJ/kg/day), sleeping heart rate (beats/min), and grip strength (kg) based on simple regression analysis

	**Activity energy expenditure **^**1 **^**(kJ/kg/day)**			**Sleeping heart rate (beats/min)**			**Grip strength (kg)**		
**Variable**	**N**	**Log B (95%CI)**^**2**^	***P***	**n**	**B (95%CI)**	***P***	**n**	**B (95%CI)**	***P***
Age (years)				304	0.07 (−0.11;0.25)	0.47	303	0.03 (−0.07;0.13)	0.57
Gestational age (weeks)	292	−0.005 (−0.007;-0.002)	0.002	299	0.15 (0.03;0.27)	0.02	298	−0.02 (−0.09;0.04)	0.49
≤28	185	Reference		189	Reference		189	Reference	
>28	107	−0.065 (−0.108;-0.023)	0.003	110	1.86 (−0.02;3.74)	0.05	110	−0.66 (−1.70;0.37)	0.21
Gravidity (number)	297			304			303		
1	117	Reference		118	Reference		117	Reference	
2	86	0.005 (−0.046;0.057)	0.84	90	1.10 (−1.09;3.30)	0.32	90	0.82 (−0.42;2.05)	0.20
≥3	94	−0.014 (−0.072;0.044)	0.64	96	1.94 (−0.21;4.10)	0.08	96	0.35 (−0.87;1.56)	0.57
Education	297			304			303		
Not completed primary school	133	Reference		133	Reference		133	Reference	
Completed primary school	164	−0.029 (−0.070;0.013)	0.17	304	2.39 (0.59;4.19)	0.01	170	1.14 (0.13;2.15)	0.03
Weight (kg)	297	−0.003 (−0.005;0.000)	0.03	304	0.20 (0.09;0.31)	< 0.001	303	0.17 (0.12;0.23)	< 0.001
Height (cm)	297	0.000 (−0.003;0.004)	0.90	304	0.07 (−0.08;0.23)	0.35	303	0.21 (0.13;0.29)	< 0.001
Body mass index (kg·m^2^)	297	−0.010 (−0.018;-0.003)	0.01	304	0.54 (0.23;0.85)	0.001	303	0.33 (0.16;0.50)	< 0.001
Arm fat area (cm^2^)	296	−0.003 (−0.005;0.000)	0.08	303	0.15 (0.03;0.27)	0.01	302	0.09 (0.02;0.16)	0.01
Arm muscle area (cm^2^)	296	0.004 (0.000;0.007)	0.07	303	0.01 (−0.16;0.17)	0.94	302	0.19 (0.10;0.28)	< 0.001

^1^Adjusted for age and cardiorespiratory fitness indicator (m/beat).

^2^Data are log-transformed beta-coefficients. The percentage difference in Activity energy expenditure associated with one unit difference in continuous exposure variables or by category is calculated as (10^B^ – 1) x 100% (95%CI).

**Table 5:** Potential predictors of activity energy expenditure (kJ/kg/day), sleeping heart rate (beats/min), and grip strength (kg) based on multiple regression analysis using backward elimination on the presented variables

	**Activity energy expenditure **^**1**^**(kJ/kg/day) Adjusted R**^**2**^**=0.17 n=291**		**Sleeping heart rate **^**2 **^**(beats/min) Adjusted R**^**2**^**=0.03 n=298**		**Grip strength **^**2**^**(kg) Adjusted R**^**2**^**=0.11 n=302**	
**Variable**	**Log B (95%CI)**^**3**^	***P***	**B (95%CI)**	***P***	**B (95%CI)**	***P***
Age (years)	−0.008 (−0.012;-0.004)	<0.001				
Gestational age (weeks)	−0.004 (−0.007;-0.001)	0.003	0.15 (0.03;0.27)	0.02		
Height (cm)					0.20 (0.11;0.28)	< 0.001
Arm fat area (cm^2^)	−0.004 (−0.008;-0.001)	0.005	0.16 (0.04;0.27)	0.01		
Arm muscle area (cm^2^)	0.006 (0.002;0.010)	0.005			0.18 (0.10;0.27)	< 0.001

^1^Adjusted for cardiorespiratory fitness indicator (m/beat).

^2^Age was forced in the model.

^3^Data are log-transformed beta-coefficients. The percentage difference in Activity energy expenditure associated with one unit difference in continuous exposure variables is calculated as (10^B^ – 1) x 100% (95%CI).

### Physical activity

BMI, weight, and gestational age were negatively associated with AEE, and AMA was positively associated with AEE (Table 4). The three adiposity measures, BMI, weight, and AFA, were negatively associated with AEE in a model with AMA, however, due to co-linearity only one variable at a time could be included in the model. Introducing BMI or weight, but not AFA or AMA, in a model with gestational age, reduced the association between AEE and gestational age. In the final multiple model for AEE (Table 5), a 10 cm^2^ higher value in AMA was associated with 14% higher AEE ((10^0.006^ – 1) × 10 × 100%). Furthermore, AEE was 10% lower for every 10 cm^2^ positive difference in AFA and 10-week difference in gestational age.

### Cardiorespiratory fitness (sleeping heart rate)

Sleeping HR was positively associated with gestational age, weight, BMI, AFA, and education in the simple analysis (Table 4). As the three adiposity measures (BMI, weight, and AFA) were not independent, only AFA was included in the final model, while BMI and weight became insignificant when gestational age was included. In the final multiple model (Table 5), a 10-week difference in gestational age was associated with a 1.5 beats/min higher sleeping HR. Furthermore, a 10 cm^2^ higher value in AFA was associated with a difference in sleeping HR of 1.6 beats/min.

### Muscular fitness (grip strength)

In the simple analysis, education and all anthropometric variables were positively associated with grip strength (Table 4). When adjusting for weight, both BMI and AFA became negatively associated with grip strength, whereas AMA was no longer associated with grip strength. In the final multiple model (Table 5), being 10 cm taller was associated with a 2.0 kg difference in grip strength. Furthermore, a 10 cm^2^ higher value in AMA was associated with 1.8 kg higher grip strength.

## Discussion

The level of physical activity among pregnant urban Ethiopian women was estimated to be low, and according to the World Health Organization classification [20] the participants would on average be classified as sedentary to lightly active. The low PAL estimated using the objective method was supported by the 24-hour physical activity recall, with large amount of sedentary activities reported.

The incorporation of both physiological and biomechanical signals in the estimation of AEE has several advantages compared to other measures of physical activity (e.g. questionnaire data). The monitor used in the current study was a small, lightweight monitor, it was easy to use in the field, and the method used for converting heart rate and acceleration into AEE provide similar estimates of AEE as indirect calorimetry in both laboratory settings [21,22] and in the free-living environment, the latter showing good agreement with the doubly labeled water method in adults from Cameroon [9]. Combined heart rate and movement sensing has previously been used in pregnant populations [10,11] but to our knowledge never among pregnant women from a low-income country. Therefore, the results presented in this paper are unique and novel, with physical activity being assessed objectively in a large sample from an understudied population. Nonetheless, AEE estimates should be interpreted with some caution, since the method is yet to be validated against doubly labeled water in pregnant African women. The main limitation of the study was the cross-sectional design. Inference on changes during pregnancy as suggested by the differences between women of different gestational age of pregnancy in this study should, therefore, be made with caution. In addition, gestational age was assessed by self-report and not verified with ultrasound, so some degree of measurement error would also exist in this variable.

Pivarnik *et al.*[23] have demonstrated that the relationship between HR and oxygen uptake (VO_2_) changes during pregnancy and will cause an overestimation of AEE at rest and light activities and an underestimation during moderate and vigorous activities. Given our study population and setting, we chose a self-paced walk test as proxy measure of cardiorespiratory fitness, which we utilized statistically to account for some of the between-individual variance in the HR-VO_2_ relationship; a step test or similar exercise test using prescribed higher intensity levels would have been too strenuous for most of the participants. Considering that the women in the present study predominantly were engaged in light intensity activities, AEE might be slightly overestimated. However, our calibration approach would remove some of the bias reported using HR-based measures during such activities, and the addition of movement sensing would further minimize this bias.

PAL is based on AEE estimated from movement and heart rate as well as the estimate of resting metabolic rate based on age, sex, height, and weight, derived for use in non-pregnant individuals [18]. Although pregnancy would most likely increase resting metabolic rate [23,24], the resting metabolic rate estimate may carry a positive bias in Africans [25]; hence PAL in this study may be estimated with a fair degree of uncertainty.

### Physical activity

The overall level of physical activity was low both when reported as AEE (kJ/kg/day) and PAL. Furthermore, the women spent most of their time in activities of sedentary and light intensity and had only a small amount of moderate-to-vigorous intensity activity. The level of physical activity among these pregnant women was also low compared to physical activity among non-pregnant women from Africa [2,26]. However, these studies are of older date and the ongoing urbanization currently occurring in many low-income countries has changed the traditional physical activity pattern [27]. When comparing physical activity among Ethiopian women in the current study with other studies using the same method, AEE was 20% lower and time engaged in moderate-to-vigorous activity was less than half compared to non-pregnant women from Cameroon [27] and even lower compared to rural Kenyan women [16]. Furthermore, AEE was 30% lower compared to a group of pregnant women from Sweden [10] and AEE of the Ethiopian women resembled inactive pregnant women more than active pregnant women from Switzerland [11]. These observations are supported by the fact that energy intake in these pregnant Ethiopian women was also found to be low (6.2 MJ/day, detailed data not shown); hence the low level of physical activity may be an appropriate adaptation to low energy intake to ensure adequate nutrition for the fetus, or may simply be explained by less physically demanding work during pregnancy.

In accordance with previous studies, AEE was negatively associated with gestational age [2,24,28,29] even though the energy cost associated with a specific activity increase continuously during pregnancy [30]. It is therefore likely that women do decrease their activity and likely also the types of activity they engage in with less vertical acceleration during the course of their pregnancy. High fat and low muscle mass in the upper arm were also associated with lower AEE, indicating a low habitual physical activity in women with less favorable body composition, which may have already existed prior to pregnancy or alternatively suggesting that body composition may relate to how a women manage to maintain her activity level when she becomes pregnant.

### Cardiorespiratory fitness (sleeping heart rate)

More advanced stage of pregnancy (gestational age) was associated with higher sleeping HR, which is in accordance with previously reported results [23]. The increase in sleeping HR during the course of pregnancy has been proposed to be caused by a decrease in parasympathic influence [31] and, therefore, may not necessarily reflect lower fitness. However, AFA was positively associated with sleeping HR and since this measure is likely unaffected by pregnancy, AFA may reflect the amount of fat present prior to pregnancy. A higher AFA during pregnancy may therefore reflect a lower cardiorespiratory fitness carried into pregnancy.

### Muscular fitness (grip strength)

AMA was positively associated with grip strength. It was expected that the amount of muscle in the upper arm was associated with muscular strength of the forearm. In general, muscular fitness was highest in women with high bodyweight, combined with low amounts of fat and high amounts of muscle. Height was independently positively associated with muscular fitness. Being taller is generally associated with longer limbs and larger hands, which by virtue of longer levers provide better working conditions for the muscles and hence enhanced ability to generate force.

In summary, concerns have been raised that pregnant women in developing countries have high levels of physical activity which can have negative effects on the child. However, our observations indicate that current levels of physical activity among pregnant urban Ethiopian women, when assessed by an objective method, are relatively low and that these women only seldom engage in activity of moderate-to-vigorous intensity. Furthermore, they do not seem to perform a large amount of physically demanding work, such as cleaning and outdoor work. Our study sample, however, only included women residing in urban Ethiopia, whose activity level may not be comparable to pregnant women living in rural areas. Low levels of physical activity have been linked to increased risk of diabetes and cardiovascular diseases in adult Cameroonian [27,32], and to increased risk of gestational diabetes mellitus as well as preeclampsia in high-income countries [33-35]. However, it is unclear whether or not the low physical activity observed in the present study carry the same increased risks or simply is an appropriate adaptation to a low energy intake. Nonetheless, it is not unlikely that both nutrient intake and physical activity need to increase in these women for optimal health and additional research is required to identify the determinants for such behavior changes. To this end, future research should focus on physical activity among pregnant women in low-income countries, its determinants, and its implications for the health of mother and child.

## Conclusion

The level and intensity of physical activity in pregnant Ethiopian women was low compared to women from low income countries as well as pregnant European women when assessed by the same method. Gestational age and degree of adiposity were both independently associated with lower activity and fitness levels, whereas, muscle mass was independently associated with higher activity and muscular fitness levels. Future research should also focus on the consequences of low physical activity during pregnancy for both mother and child.

## Abbreviations

AEE: Activity energy expenditure; AFA: Arm fat area; AMA: Arm muscle area; BMI: Body mass index; CI: Confidence interval; HR: Heart rate; IQR: Inter quartile range; MET: Metabolic equivalence of task; MUAC: Mid-upper-arm circumference; PAL: Physical activity level; SD: Standard deviation; TSF: Triceps skinfold thickness.

## Competing interests

This project was funded in whole by DANIDA (104.Dan.8-1207) and the Danish Council for Strategic Research – Programme Commission on Food and Health, the Danish Council for Independent Research, Medical Sciences. None of the authors have any conflict of interest to declare.

## Authors’ contributions

TG, GA, PK, SB and HF conceived the study. MH, SK, TG, GA and PK coordinated the implementation of the study. MH and SK analyzed the data and wrote the first draft of the manuscript. MH, SK, DFJ, SB and HF contributed to the interpretation of results. All authors commented on drafts and approved the final version. HF is guarantor of the paper.

## Authors’ information

First authorship: Mads F Hjorth and Stine Kloster.

## Pre-publication history

The pre-publication history for this paper can be accessed here:

http://www.biomedcentral.com/1471-2393/12/154/prepub
